# Identifying DASH Adherence Patterns and Their Association With Dietary Behaviors Among Adults With Hypertension

**DOI:** 10.1002/nur.70078

**Published:** 2026-05-04

**Authors:** Youran Lee, Melissa C. Kay, Anushka Palipana, Sandy Askew, Miriam B. Berger, Hailey N. Miller, Gary G. Bennett, Qing Yang

**Affiliations:** ^1^ Duke University School of Nursing, Durham, NC, USA (Currently in University of Michigan School of Nursing Ann Arbor Michigan USA; ^2^ School of Medicine Wake Forest University Winston‐Salem North Carolina USA; ^3^ Duke Global Health Institute Duke University Durham North Carolina USA; ^4^ Johns Hopkins School of Nursing Baltimore Maryland USA; ^5^ Department of Psychology and Neuroscience Duke University Durham North Carolina USA; ^6^ Trinity College of Arts & Sciences Duke University Durham North Carolina USA

**Keywords:** diet, dietary adherence, dietary approaches to stop hypertension, eating behavior, hypertension

## Abstract

The Dietary Approaches to Stop Hypertension (DASH) diet is effective in lowering blood pressure yet adherence to DASH remains low. Intuitive eating, a behavior that emphasizes responsiveness to hunger and satiety cues, may influence DASH adherence but has not been well studied among adults with hypertension. This study examined DASH adherence subgroups and their association with dietary behaviors using data from the Nourish U.S. based randomized controlled trial. A cross‐sectional secondary analysis of baseline data from 301 participants was conducted. DASH adherence was assessed using the Mellen Index. Dietary behaviors were measured with the Intuitive Eating Scale‐2 (IES‐2). Latent class analysis (LCA) was used to identify DASH adherence subgroups, and subgroup differences in dietary behaviors and clinical outcomes were analyzed. Overall, DASH adherence was low (mean = 3.13 ± 1.35). LCA identified three DASH adherence subgroups: low adherence (44.2%), high adherence (41.2%), and mixed‐pattern adherence (14.6%). Subgroups significantly differed in IES‐2 subscales, including unconditional permission to eat (*p* < 0.0001), reliance on hunger and satiety cues (*p* = 0.0487), and body‐food choice congruence (*p* < 0.0001). The low‐adherence group scored higher on hunger cues, while the high‐adherence group scored higher on body‐food choice congruence. These findings highlight the value of examining overall dietary patterns rather than a single DASH score, as patterns better capture interactions among foods and nutrients that influence diet quality and health. Each subgroup's distinct demographic and behavioral characteristics further emphasize tailored DASH diet guidance which could lead to better cardiovascular outcomes.

**Patient or Public Contribution:**

301 adults with hypertension contributed to this study by providing baseline data as part of the Nourish randomized controlled trial (ClinicalTrials.gov: NCT03875).

## 1 Introduction

Nearly one in two adults in the United States (US) has hypertension (Ostchega et al. 2020), a leading risk factor for stroke, kidney disease, heart disease, and premature mortality (Fuchs and Whelton 2020; Writing Committee et al. 2025). The Dietary Approaches to Stop Hypertension (DASH) diet is a well‐established dietary pattern to prevent and treat hypertension (U.S. Department of Agriculture 2020). The DASH diet emphasizes reduced sodium intake, increased consumption of fruits and vegetables, whole grains, and consumption of low‐fat dairy products; it limits red meats, sugar, and refined carbohydrates (Ndanuko et al. 2017). Adopting DASH can significantly lower both systolic and diastolic blood pressure (BP), thereby reducing the risk of cardiovascular events (Guo et al. 2021; Filippou et al. 2020; Soltani et al. 2020).

Despite effectiveness of the DASH diet on managing hypertension, national data show adoption at the population level is suboptimal. In fact, about 1.5% of adults with hypertension fully adhere to the DASH diet, and fewer than 20% meet at least half of the recommended guidelines (Kwan et al. 2013; Mellen et al. 2008). Numerous clinical trials have investigated various interventions to improve adherence (Filippou et al. 2020; Kwan et al. 2013; Tan et al. 2024). Controlled feeding trials, in which dietary conditions are tightly regulated, demonstrate a high adherence rate; however, behavioral trials that mirror real‐world settings consistently report significantly lower adherence levels. This discrepancy may be due to the substantial behavioral changes required to adopt the DASH diet, including shifts in individual consumption patterns and cooking methods (Tyson et al. 2023). Additionally, adherence often varies across components of the DASH diet, making it difficult to assess overall dietary compliance (Lutski et al. 2025). Studies have examined correlations in adherence across nutrient groups which suggest that individuals may follow certain components more closely than others due to underlying dietary behaviors or preferences (Fung 2008). Such findings underscore the importance of examining patterns of dietary consumption rather than focusing on individual nutrients alone, as dietary patterns may better capture interactions among foods and nutrients and be more predictive of chronic disease outcomes (Shang et al. 2023).

Adherence to the DASH diet may also be related to underlying dietary behaviors, which comprise the individual's decisions and actions regarding their diet (e.g., what, when, why, and how they eat) (Marijn Stok et al. 2018). Intuitive eating, an approach that promotes eating in response to internal hunger and satiety cues rather than external dietary rules, has gained increasing attention for its associations with healthier eating patterns, higher diet quality, and more positive relationships with food and body image (Bruce and Ricciardelli 2016). Emerging evidence suggests that intuitive eating is a multidimensional construct. Specific subscales of the Intuitive Eating Scale (IES) may relate to diet quality more strongly than the total score. For example, the “Body–Food Choice Congruence” subscale is consistently associated with higher diet quality, whereas the “Unconditional Permission to Eat” subscale often demonstrates neutral or negative associations (Lopez et al. 2023; Sire et al. 2025). These findings indicate the importance of examining intuitive eating at the subscale level rather than relying solely on overall scores. Systematic reviews further suggest that intuitive eating–based interventions may improve diet adherence by enhancing responsiveness to internal cues and reducing maladaptive behaviors such as emotional or binge eating (Hensley‐Hackett et al. 2022). Although intuitive eating has been linked to higher diet quality and favorable cardiometabolic markers (e.g., lipid profiles) in observational studies, relatively little evidence directly connects intuitive eating with blood pressure outcomes in adults with diagnosed hypertension, or with adherence to evidence‐based dietary patterns such as DASH (Eaton et al. 2024; Teas et al. 2022). Moreover, there have been few evaluations of interventions that promote intuitive eating principles. Notably, much of the existing literature has been conducted in generally healthy or community‐based samples rather than clinical hypertension cohorts (e.g., community older adults and general adult populations) (Teas et al. 2022; Van Dyke and Drinkwater 2022; Sire et al. 2025). This represents a critical gap, as empowering individuals to adopt positive, sustainable eating behaviors may be key to enhancing adherence to DASH dietary guidelines in real‐world settings among at risk populations.

To mitigate this gap, the present study used data from Nourish, two‐arm, 12‐month randomized controlled trial aimed at improving DASH adherence among adults with hypertension, to examine the impact of intuitive eating behaviors on DASH adherence patterns through person‐centered approach. The specific aims of this study were to (1) describe DASH adherence in adults with hypertension and identify distinct DASH adherence subgroups using latent class analysis (LCA); and (2) examine differences in intuitive eating behaviors and systolic and diastolic BP clinical outcomes among DASH adherence subgroups.

## 2 Methods

### 2.1 Study Design and Samples

This cross‐sectional study was an exploratory secondary analysis of baseline data from the Nourish dataset, described in‐detail elsewhere (Miller et al. 2025; Miller et al. 2021). Briefly, Nourish was a two‐arm, 12 month randomized controlled efficacy trial conducted in the United States (North Carolina) that leveraged commercially available smartphone applications and evidence‐based behavior to change principles to improve adherence to the DASH eating pattern among adults with hypertension (Miller et al. 2021). For the current study, we used Nourish baseline data only (*n* = 301).

### 2.2 Measures

#### 2.2.1 Demographic Characteristics

Collected demographic characteristics included age, gender, race, ethnicity, living with a partner, and socioeconomic status (i.e., education level, employment, income, insurance).

#### 2.2.2 Dietary Behaviors

Dietary behaviors were measured by the Intuitive Eating Scale‐2 (IES‐2) (Tylka and Kroon Van Diest 2013), a 23‐item tool designed to capture the essence of intuitive eating. IES‐2 demonstrated good internal consistency reliability (Cronbach's *α* = 0.87 [women], 0.89 [men]) and convergent validity, showing strong correlations with the original IES total score (*r* = 0.87 [women], 0.91 [men]) and corresponding subscales (*r* = 0.86–0.95) (Tylka and Kroon Van Diest 2013). The questionnaire is divided into four subscales: (1) the “unconditional permission to eat” subscale (6 items), which measures individual attitudes toward food and eating in a nonrestrictive manner; (2) the “eating for physical rather than emotional reasons” subscale (8 items), which measures emotional triggers for eating; (3) the “reliance on hunger and satiety cues” subscale (6 items), which measures recognition and response to physical hunger and fullness signals; and (4) the “body‐food choice congruence” subscale (3items), which measures how food choices align with the body's needs and health‐consciousness. The IES‐2 utilizes a 5‐point Likert‐type scale (1 = *strongly disagree* to 5 = *strongly agree*) and includes seven negatively worded items meant for reverse scoring to ensure accuracy in reflecting intuitive eating tendencies (Tylka 2006). Higher scores on the IES‐2 and its subscales indicate a greater tendency toward intuitive eating, meaning that the individual is more likely to eat based on internal cues and to choose foods that are congruent with their body's needs. Lower scores suggest a tendency towards non‐intuitive eating behaviors, such as eating in response to emotional cues, ignoring hunger and satiety signals, and following rigid dietary rules. The total score ranges from 23 to 115, with the average score ranging from 1 to 5 after dividing by the total number of questions.

#### 2.2.3 DASH Adherence

Dietary intake data were collected using the web‐based Automated Self‐Administered 24 h (ASA24®) recall tool developed by the National Cancer Institute, which provides comprehensive nutrient data on all foods and beverages consumed within the past 24 h (National Cancer Institute 2025). ASA24® guides respondents through a structured, self‐administered 24 h recall using a modified version of the USDA Automated Multiple‐Pass Method (AMPM), allowing researchers to collect standardized, detailed intake data on foods and beverages consumed during the previous day (Subar et al. 2012). At baseline, each participant was asked to complete two 24 h dietary recalls using the ASA24, one on a weekday and one on a weekend. Dietary recall data was considered complete if the participant tracked ≥ 600 calories in 1 day, as this is typically the minimum level of calories needed to determine whether the data represents a complete day of tracking (Subar et al. 2012).

The Mellen index was used to assess the dietary recall data for DASH adherence. The Mellen index operationalizes DASH adherence using nine nutrient‐based targets derived directly from the nutrient goals of the original DASH feeding trials (total fat, saturated fat, magnesium, calcium, potassium, sodium, cholesterol, fiber, and protein) (Mellen et al. 2008). Each nutrient is standardized to total calorie intake making it less sensitive to variation in food choices, fostering comparisons across demographic groups (Miller et al. 2013). Each of the 9 components is given a score of 0 = *not meeting the goal*, 0.5 = *meeting an intermediate goal*, or 1 = *fully achieving the goal* (see Supplementary Table A). Individual nutrient scores are summed to calculate a total DASH adherence score; the range is from 0 to 9, with higher scores indicating greater adherence to the DASH eating pattern (Mellen et al. 2008). The Mellen Index has demonstrated validity across multiple health outcomes (Julián‐Serrano et al. 2022), and because the digital intervention delivered nutrient‐specific dietary goals, the nutrient‐based index provided the most conceptually aligned and internally consistent measure of DASH adherence.

#### 2.2.4 Clinical Status

Baseline BP was included as a clinical status variable in this secondary analysis of baseline Nourish data. In the Nourish trial, study visits (including the baseline/randomization visit) were conducted remotely via Zoom, and BP was measured during these staff‐facilitated online visits. All participants were provided with an Omron series 3 digital BP monitor for at‐home measurement during study visits. BP was measured three times according to the study's remote BP measurement protocol, and the average of the last two readings was used for analysis (Miller et al. 2021).

#### 2.2.5 Ethical Consideration

The parent Nourish trial was approved by the Duke University IRB, and all participants provided informed consent. The current secondary data analysis was also reviewed and approved by the Duke University IRB, with informed consent waived due to the use of previously collected/de‐identified data.

### 2.3 Statistical Analyses

Analyses were conducted using SAS 9.4 (SAS Institute Inc, Cary, NC). Descriptive statistics were used to detail the sample characteristics (*N* = 301) and all study measures. Nondirectional inferential statistical tests were conducted at a 0.05 significance level, which was not adjusted for multiple tests due to the exploratory nature of the analysis.

**Figure 1:**
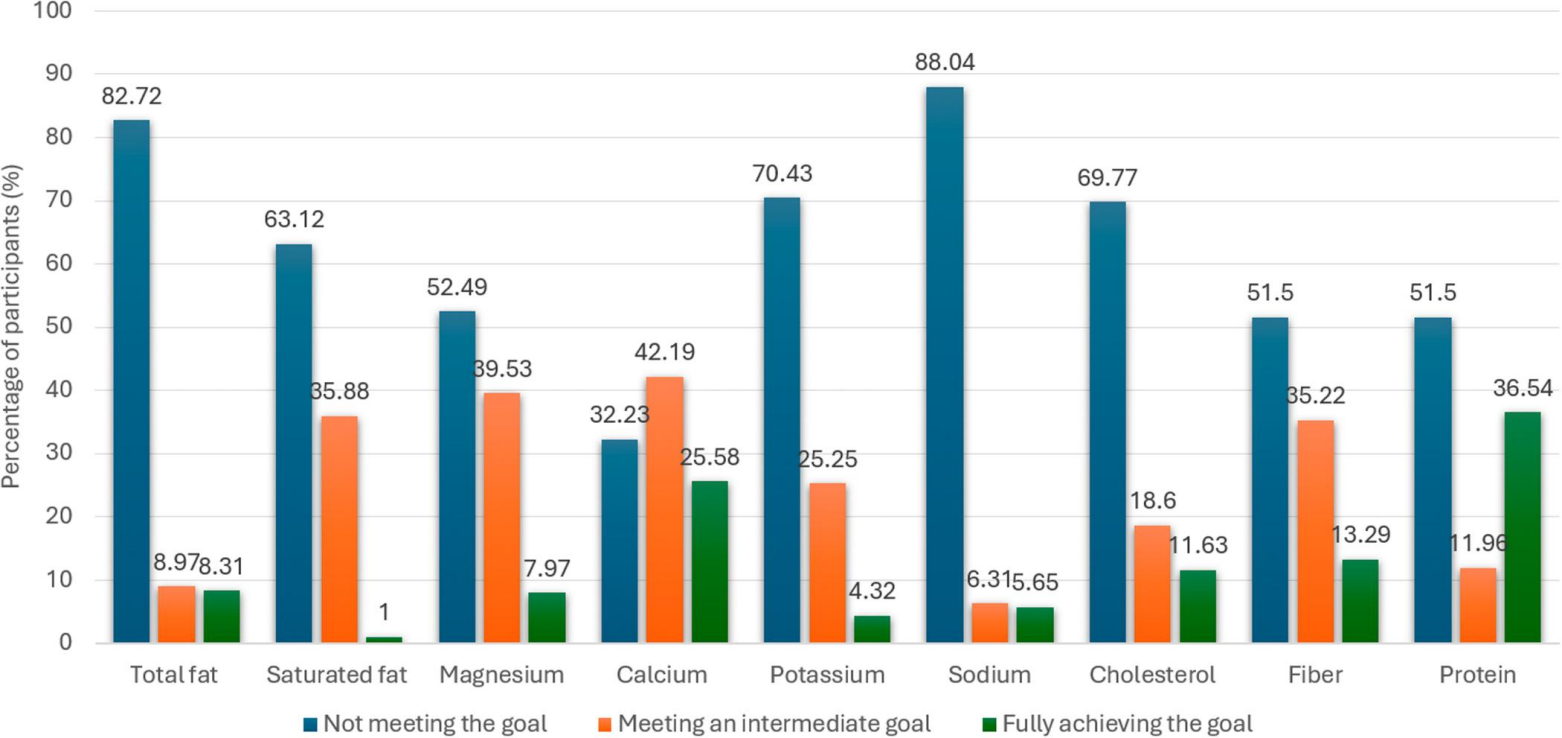
Adherence to DASH dietary components as measured by the Mellen Index among participants in the Nourish trial (*n* = 301).

Bar charts were utilized to illustrate the adherence levels to the nine nutritional components of the Mellen index, with cut‐off points indicating whether individuals did not meet the goal, met the intermediate target, or fully met the dietary recommendations (Figure 1). As most participants did not meet the goal, we combined the categories to “not meeting the goal” and “meeting or exceeding the intermediate goal.” The dichotomized nutrition components adherence measure was then used in LCA.

LCA assumes that a population consists of heterogeneous subgroups of individuals who share similar characteristics. It is a person‐centered modeling technique used to identify latent subgroups (classes) based on an unobserved construct (Lanza and Rhoades 2013). Therefore, LCA was performed to identify subgroups with distinct DASH diet adherence patterns. We tested a sequence of LCA models that included each of the nine nutrition components. Each model differed in the specified number of latent classes (subgroups) to determine the best‐fitting model and optimal subgroup sizes.

LCA models with two to five classes were evaluated, and the final model was selected based on goodness‐of‐fit indices, simplicity, and clinical interpretability to ensure effective representation of distinct diet adherence profiles. Model fit indices included log‐likelihood, Akaike information criterion (AIC), Bayesian information criterion (BIC), and Sample‐Size Adjusted BIC (SBIC). Entropy values can range from 0 to 1, with higher scores indicating a greater degree of subgroup (class) separation (Lanza and Rhoades 2013). A recommended entropy value greater than 0.80 indicates strong classification accuracy, with higher entropy reflecting more precise assignment of individuals to profiles. Additionally, lower log‐likelihood, AIC, BIC, and SBIC scores generally signify better model fit (Weller et al. 2020).

Differences in dietary behaviors and clinical outcomes across subgroups were explored. Pearson's chi‐square tests were conducted for categorical variables (socioeconomic status), and analysis of variance (ANOVA) techniques were employed for continuous variables (dietary behaviors). Tukey's honestly significant difference (HSD) test was used for post‐hoc comparisons to identify specific inter‐subgroup differences.

## 3 Results

### 3.1 Sample Characteristics

Table 1 presents the descriptive demographic characteristics of the sample. Most individuals were over 60 years old (38.9%), female (65.1%), non‐Hispanic/Non‐Latinx White (53.5%), and had a postgraduate degree (43.7%). The majority lived with a partner (63.8%) and nearly half worked full‐time (49.2%). Many reported an annual income of $100,000 or more (37.1%), and most had private insurance (66.0%). The subgroup and total scores of the IES‐2 fell within the medium range, indicating a moderate level of intuitive eating behaviors (Tylka and Kroon Van Diest 2013). The total IES‐2 scale score was 3.26 (SD = 0.50). The average systolic BP was 127.86 mmHg (SD = 13.91), and the average diastolic BP was 82.20 mmHg (SD = 9.50). The overall DASH score was 3.13 (SD = 1.35). As presented in Table 1, subgroup differences in baseline characteristics were generally not statistically significant; education was the only characteristic that differed significantly across subgroups. Descriptively, subgroup 3 (relatively high adherence) included slightly more participants who were older, subgroup 2 (low adherence) included slightly more participants who were non‐Hispanic Black/African American, and participants with lower levels of education. Subgroup 1 (mixed pattern adherence) included slightly more participants with higher incomes and fewer participants with Medicare. Although not statistically different, most of those variables demonstrated effect sizes greater than 1, suggesting meaningful differences across subgroups.

**Table 1:** Baseline Demographic Characteristics of Participants from the Nourish Study (*n* = 301).

Characteristic	Total (*n* = 301)	Subgroup 1 (Mixed pattern adherence) (*N* = 44)	Subgroup 2 (Low adherence) (*N* = 133)	Subgroup 3 (Relative high adherence) (*N* = 124)	R‐square	*p‐*value
Age (mean ± sd)	54.40 (13.49)	52.50 ± 12.23	52.97 ± 13.08	56.60 ± 13.91	0.0191	0.0564
Age (*n*, %)	301	44	133	124	Effect size (Cramer's V)	301
Less than 40 years old	49 (16.28)	9 (20.45)	23 (17.29)	17 (13.71)	0.1189**	0.2032
Age 40 to 49	65 (21.59)	12 (27.27)	33 (24.81)	20 (16.13)		
Age 50 to 59	70 (23.26)	9 (20.45)	33 (24.81)	28 (22.58)		
More than 60 years old	117 (38.87)	14 (31.82)	44 (33.08)	59 (47.58)		
Sex (*n*, %)	301	44	133	124		301
Male	105 (34.88)	16 (36.36)	43 (32.33)	46 (37.10)	0.0479	0.7077
Female	196 (65.12)	28 (63.64)	90 (67.67)	78 (62.90)		
Race and ethnicity (*n*, %)	301	44	133	124		301
Non‐Hispanic White	161 (53.49)	24 (54.55)	64 (48.12)	73 (58.87)	0.1037**	0.3717
Non‐Hispanic Black/African American	93 (30.90)	12 (27.27)	49 (36.84)	32 (25.81)		
Hispanic/Latinx	22 (7.31)	2 (4.55)	10 (7.52)	10 (8.06)		
Other races and unreported	25 (8.31)	6 (13.64)	10 (7.52)	9 (7.26)		
Education (*n*, %)	300	44	132	124		300
Less than a 4‐year college degree	71 (23.67)	5 (11.36)	43 (32.58)	23 (18.55)	0.1378**	0.0225*
College graduate	98 (32.67)	17 (38.64)	39 (29.55)	42 (33.87)		
Postgraduate degree	131 (43.67)	22 (50.00)	50 (37.88)	59 (47.58)		
Living with partner (*n*, %)	301	44	133	124		301
No	109 (36.21)	15 (34.09)	52 (39.10)	42 (33.87)	0.0534	0.6507
Yes	192 (63.79)	29 (65.91)	81 (60.90)	82 (66.13)		
Employed (*n*, %)	301	44	133	124		301
Full‐time	148 (49.17)	23 (52.27)	68 (51.13)	57 (45.97)	0.0732	0.5214
Part‐time	36 (11.96)	3 (6.82)	19 (14.29)	14 (11.29)		
Not doing paid work	117 (38.87)	18 (40.91)	46 (34.59)	53 (42.72)		
Income (*n*, %)		42	129	120		291
Less than $25,000	28 (9.62)	3 (7.14)	18 (13.95)	7 (5.83)	0.1002**	0.2116
$25,000‐$74,999	118 (40.56)	15 (35.71)	51 (39.53)	52 (43.33)		
Over $75,000	145 (49.82)	24 (57.14)	60 (46.51)	61 (50.83)		
Insurance (*n*, %)	291	44	132	124		300
Private	198 (66.00)	30 (68.18)	90 (68.18)	78 (62.90)	0.1556**	0.1505
Medicaid	17 (5.67)	4 (9.09)	9 (6.82)	4 (3.23)		
Medicare	71 (23.67)	6 (13.64)	27 (20.45)	38 (30.65)		
Tricare or Veteran Administration Benefits	4 (1.33)	1 (2.27)	3 (2.27)	0 (0.00)		
None	8 (2.67)	3 (6.82)	2 (1.52)	3 (2.42)		
Others	2 (0.67)	0 (0.00)	1 (0.76)	1 (0.81)		
Clinical variable (mean ± sd)					R‐square	*F‐*value(*p*‐value)
Systolic blood pressure	127.86 ± 13.91	124.30 ± 14.42	127.15 ± 14.69	129.89 ± 12.58	0.0196	2.98 (0.0524)
Diastolic blood pressure	82.20 ± 9.50	81.75 ± 9.51	82.41 ± 10.21	82.14 ± 8.75	0.0005	0.08 (0.9205)

* Significant at *p* < 0.05.

** *R* ^2^ ≥ 0.10 indicates meaningful effect size.

Intake for most of the DASH nutrition components did not meet recommended levels (Figure 1). The nutrients with the highest adherence rates for meeting the target goal were protein (36.54%), calcium (25.58%), and fiber (13.29%), and lowest for saturated fat (1.00%), potassium (4.32%), and sodium (5.65%).

#### 3.1.1 Patterns of DASH Adherence

Table 2 provides the entropy and fit indices for the datasets, highlighting the best‐fitting model results. While the 4‐class model exhibited slightly better fit indices (lowest BIC and CAIC) and similarly high entropy (0.85), the 3‐class model was deemed the optimal solution because it offered the most coherent and theoretically sound explanation of the latent structure, balancing statistical fit with conceptual clarity (Collins and Lanza 2009). Although the log‐likelihood, AIC, and sample‐size adjusted BIC (SBIC) continued to decrease with the addition of more latent profiles, there was a noticeable leveling‐off after the introduction of the 3‐class model. This further supported the selection of the 3‐class model as the most parsimonious and best‐fitting solution.

**Table 2:** Latent Class Analysis: Model fit Indices for the Specified Number of Classes.

Classes	*LogLik	*AIC	*BIC	*CAIC	Adjusted BIC	df	Entropy
2	−1498.28	511.62	582.06	601.06	521.80	492	0.85
3	−1439.77	413.80	521.31	550.31	429.33	482	0.83
4	−1404.38	362.83	508.40	547.40	384.72	472	0.85
5	−1392.55	360.15	541.80	590.80	386.40	462	0.85

Abbreviations: AIC = Akaike Information Criterion, BIC = Bayesian Information Criterion, CAIC = Consistent Alkaike Information Criterion, df = degrees of freedom, LogLik = Log‐Likelihood.

**Figure 2:**
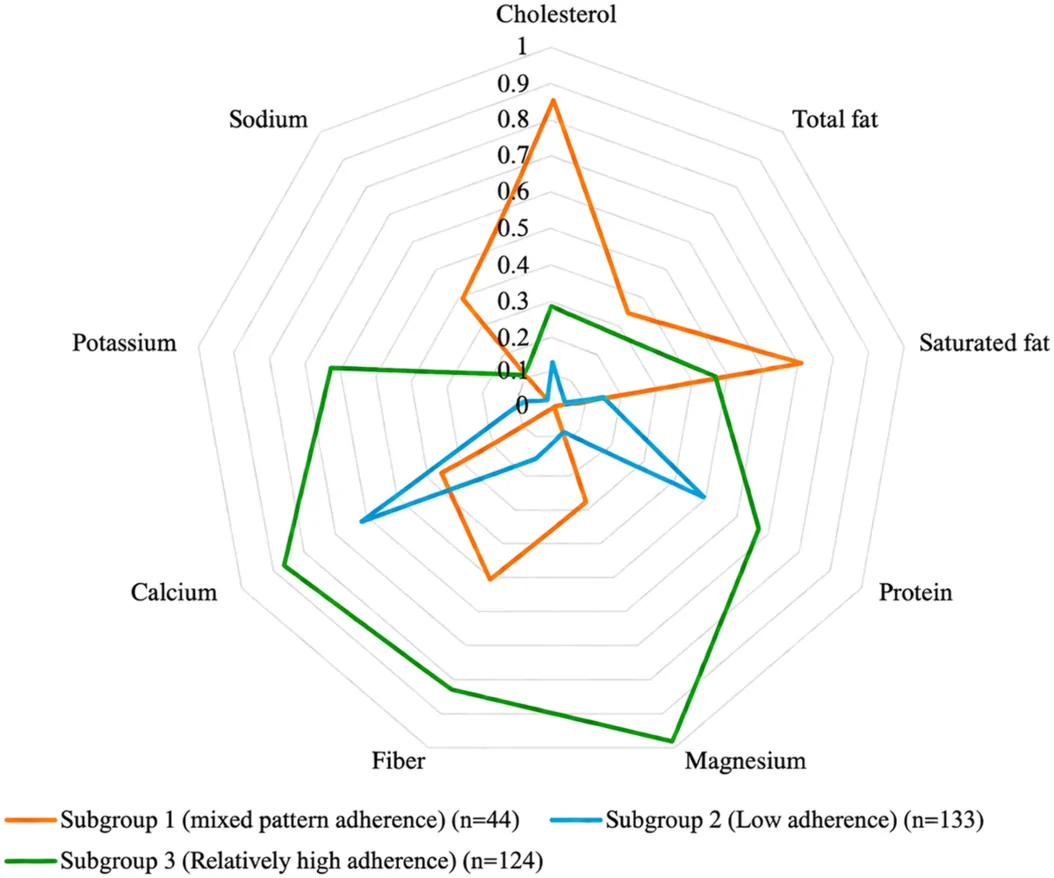
Identified Dietary Adherence Subgroups Based on Latent Class Analysis among participants in the Nourish trial (*N* = 301).

Figure 2 shows three distinct dietary adherence subgroups through radar plot based on their compliance with the DASH guidelines using the Mellen Index. Subgroup 1 (mixed pattern adherence) comprised of 44 participants (14.62%) and was characterized by relatively high adherence to cholesterol, saturated fat, but low adherence to protein and potassium. Subgroup 2 (low adherence), the largest subgroup with 133 participants (44.19%), exhibited low adherence to almost all nutritional categories. Subgroup 3 (relative high adherence), comprised of 124 participants (41.19%), exhibited relative higher adherence to all nutritional categories except for sodium where adherence is low for everyone.

Table 3 presents the mean scores and standard deviations (SD) for the intuitive eating subscales, DASH Mellen score, and BP across the total sample (*N* = 301) and among the three identified subgroups. There were significant differences in the unconditional permission to eat subscale, reliance on hunger and satiety cues subscale, and body‐food choice congruence subscale among subgroups (Table 3). However, no significant differences were found in eating for physical rather than emotional reasons subscale and total intuitive eating scale. BP, including average systolic and diastolic BP, did not show significant differences across the subgroups. The average systolic BP ranged from 124.30 to 129.89 mmHg, and the average diastolic BP ranged from 81.75 to 82.41 mmHg across the total sample. Subgroup 1 (mixed pattern adherence) had the lowest systolic BP, and subgroup 3 had the highest; however, these differences were not statistically significant (Table 1).

**Table 3:** Differences in Intuitive Eating Scores and Clinical Indicators Across DASH Adherence Subgroups among participants in the Nourish trial.

	Total (*N* = 301)	Subgroup 1 (Mixed pattern adherence) (*N* = 44)	Subgroup 2 (Low adherence) (*N* = 133)	Subgroup 3 (Relatively high adherence) (*N* = 124)	F (*p*‐value)
Intuitive eating	Mean ± SD	Mean ± SD	Mean ± SD	Mean ± SD	
Unconditional Permission to Eat subscale	3.08 ± 0.60	3.25 ± 0.56	3.20 ± 0.53	2.88 ± 0.63	11.99 (< 0.0001)*
Eating for Physical Rather than Emotional Reasons subscale	3.36 ± 0.89	3.57 ± 0.82	3.24 ± 0.94	3.41 ± 0.85	2.59 (0.0767)
Reliance on Hunger and Satiety Cues subscale	3.20 ± 0.75	3.37 ± 0.73	3.18 ± 0.72	3.17 ± 0.78	3.05 (0.0487)*
Body‐Food Choice Congruence subscale	3.51 ± 0.78	3.55 ± 0.93	3.29 ± 0.73	3.74 ± 0.72	11.48 (< 0.0001)*
Total Intuitive Eating Score	3.26 ± 0.50	3.43 ± 0.46	3.22 ± 0.51	3.25 ± 0.49	2.11 (0.0990)

* Significant at *p* < 0.05.

## 4 Discussion

This study aimed to identify patterns of adherence to the DASH diet adherence as measured by the Mellen Index, and to examine the association between intuitive eating behaviors and adherence patterns among adults with hypertension. To our knowledge, this is the first study to characterize DASH diet adherence patterns specifically among individuals with hypertension, and this unique perspective providing the importance of examining patterns of nutrition consumption, as dietary patterns may better capture interactions among foods and nutrients and be more predictive of chronic disease outcomes. Overall, adherence to DASH diet was low, with particularly poor adherence to sodium recommendations. These findings are consistent with previous literature (Kwan et al. 2013; X. Theodoridis et al. 2023). More importantly, we identified three distinct DASH adherence subgroups. In addition to subgroup 2 (low adherence) and subgroup 3 (relatively high adherence), we found a group with mixed pattern adherence. These findings underscore the importance of examining dietary patterns instead of focusing solely on the overall DASH score, since dietary patterns better capture the synergistic relationships among foods and nutrients that shape overall diet quality and health effects. We also found that each subgroup has unique demographic and behavioral profiles, which underscore the need for targeted interventions to improve DASH adherence within adults with hypertension.

We identified three subgroups with different DASH adherence patterns. Subgroup 2 (low adherence), which includes 44% of the sample, showed the lowest adherence across almost all nutrition, including cholesterol, saturated fat, total fat, magnesium, fiber, potassium, and sodium. In contrast, subgroup 3, comprising 41% of the sample, exhibited relatively high adherence to all nutrition except for sodium. Subgroup 1 (mixed pattern adherence), representing 15% of the sample, exhibited a mixed pattern, higher adherence to cholesterol and saturated fat, but low adherence to protein and potassium. Majority of the samples either show consistently higher or lower adherences across different nutrients, suggesting that individuals who pay attention to their diet tend to do so comprehensively, rather than selectively focusing on specific nutrients. For these individuals, effective interventions may need to focus on raising overall nutrition awareness and addressing underlying lifestyle or motivational factors that influence multiple aspects of eating behavior simultaneously, which has been the focus of previous interventions (Al‐Salmi et al. 2022; Batista de Lima and Eleuteri 2021). However, the relatively small subgroup 1 (mixed‐pattern adherence) reveals more nuanced dietary behaviors. In this subgroup, inverse association between certain nutrients specifically (e.g., high fiber and potassium with low saturated fat) suggests a potential trade‐off between healthy and unhealthy food categories. These patterns emphasize the complexity of real‐world dietary behaviors and underscore the importance of not only examining total adherence scores but also understanding how adherences to specific nutrients cluster together. Although we did not assess specific food group intake, it is plausible that individuals consumed more meat, and those with high potassium and fiber intake consumed more fruits and vegetables (National Institute on Aging 2022). Overall, these distinctions emphasize the need for more tailored dietary interventions that reflect subgroup‐specific needs, lifestyles, tendencies to promote healthier diet behaviors and better hypertension management.

The diet adherence subgroups also demonstrated distinct demographic and behavioral characteristics. Subgroup 3 (relatively high adherence) showed a pattern of having a higher proportion of participants who were non‐Hispanic White, living with a partner, and earning a higher income, a finding in alignment with existing literature on dietary adherence (Odoms‐Young and Bruce 2018; Theodoridis et al. 2023). This group potentially signifying a greater ability to purchase high‐quality foods like fresh produce, lean proteins, and whole grains that tend to be more expensive (Lewis et al. 2023). Subgroup 2 (low adherence) showed limited awareness of nutritional quality, which aligns with their overall lower levels of education. This finding may reflect a broader relationship between structural barriers and discrimination, as historical discrimination, xenophobia, and bias have contributed to historically underserved populations having fewer opportunities to approach resources such as healthy food accessibility, thus perpetuating disparities in diet and health outcomes (Odoms‐Young and Bruce 2018). Previous research has shown notable differences in diet preferences and adherence across racial and ethnic groups. For example, African Americans increased their consumption of DASH‐recommended foods after receiving DASH dietary counseling (Epstein et al. 2012) but reported lower overall adherence to the DASH eating plan compared to their White counterparts and continued to consume less fruit and more meat, sweets, and fat. These findings are consistent with our study results, which showed that the low diet adherence group had the highest proportion of Black/African American participants (Epstein et al. 2012). These results suggest that effective dietary interventions may need to be tailored to address structural and socioeconomic barriers, such as improving access to affordable healthy foods and providing culturally relevant dietary education. For example, the lower adherence group may benefit from community‐based nutrition and literacy programs that improve access to affordable healthy foods, higher adherence group could benefit from targeted interventions to reduce sodium intake, and the mixed pattern may require strategies to increase protein and potassium consumption.

In our study, significant differences were observed among the three IES subscales across the DASH adherence subgroups. Specifically, subgroup 2 (low adherence) scored highest on the *Unconditional Permission to Eat* subscale, which indicates greater flexibility and less restriction in eating behaviors without labeling foods as “good” or “bad” (Tylka and Kroon Van Diest 2013). Prior studies suggest that this flexibility can be a double‐edged sword; while it may reduce psychological rigidity around food, it is often associated with poorer dietary quality, higher intake of added sugars, and lower adherence to structured dietary plans such as the DASH diet (Lopez et al. 2023; Sire et al. 2025; Hensley‐Hackett et al. 2022). Our findings support this interpretation, showing that excessive dietary freedom without nutritional awareness may hinder adherence to recommended nutrient targets. Therefore, interventions should aim to balance self‐permission with nutritional mindfulness to promote healthier eating among adults with hypertension.

The subgroup 2 (low adherence) scored higher on the *Reliance on Hunger and Satiety Cues* subscale, indicating individuals have a stronger reliance on internal cues rather than external dietary guidelines. This intuitive approach, while potentially fostering body awareness, may conflict with the structured and rule‐based nature of the DASH diet, which emphasizes specific nutrient targets and serving patterns. Conversely, individuals in subgroup 3 (relatively high adherence**)** scored lowest on this subscale, suggesting that they may place less emphasis on internal cues and greater focus on meeting specific dietary goals. This trend may partly reflect the higher proportion of older adults in this group, as physiological changes associated with aging, such as reduced metabolic rate and altered appetite regulation, can diminish hunger and fullness awareness (Roberts and Rosenberg 2006). These findings underscore the importance of tailoring dietary interventions to individuals’ cue responsiveness, helping those who rely heavily on internal cues to integrate structured dietary principles while supporting others to remain attuned to bodily signals.

Lastly, participants in subgroup 3 (relatively high adherence) scored the highest on *Body–Food Choice Congruence* subscale, whereas subgroup 2 (low adherence) scored the lowest. Since this subscale reflects the degree to which individuals make food choices aligned with their physiological needs rather than external pressures or emotional cues (Tylka and Kroon Van Diest 2013), it suggests that individuals with greater adherence to the DASH diet may be more likely to intentionally select foods based on nutritional value rather than convenience or cravings. Consistent with prior research, this component has been positively associated with higher overall diet quality, as individuals who make food choices congruent with bodily needs tend to consume more vegetables, fruits, and whole grains and fewer added sugars and saturated fats (Lopez et al. 2023; Sire et al. 2025; Jackson et al. 2022). These findings align with prior evidence linking body–food choice congruence with improved dietary quality and self‐regulation. Integrating this concept into DASH diet education could enhance adherence by helping individuals internalize healthy food choices rather than relying solely on external dietary rules.

### 4.1 Implications

This study highlights (a) distinct DASH dietary adherence patterns linked to specific dietary behaviors, offering practical insights for improving health outcomes in adults with hypertension who require lifelong dietary management; and (b) underscores the necessity of addressing the diverse and multifaceted needs of these populations to effectively change dietary behaviors. Future interventions focusing on educating patients having chronic conditions about the benefits of positive dietary behaviors, such as intuitive eating, to boost motivation, which is important for dietary adherence, may be more effective in adopting the DASH diet (Al‐Salmi et al. 2022; Batista de Lima and Eleuteri 2021). Providing the same DASH education to all patients is often inefficient, as dietary challenges vary across individuals. For example, one subgroup may regularly consume vegetables but avoid dairy, requiring education on low‐fat dairy alternatives, while another subgroup may struggle with excessive sodium intake, benefiting more from counseling on salt‐reduction cooking strategies. By identifying these subgroups through LCA, tailored interventions can be developed, supporting a precision nutrition approach. Furthermore, the latent classes identified by LCA can be translated into simple screening tools—using just a few key questions about fruit and vegetable intake, processed food consumption, or dairy use—allowing clinicians to quickly classify patients into adherence groups and identify high‐risk populations in practice. Beyond clinical application, LCA advances knowledge by uncovering behavioral clustering, illustrating how dietary habits co‐occur rather than merely highlighting differences in average adherence. This provides deeper insights into the complex interplay between behaviors, diet, and health outcomes.

### 4.2 Limitations

This study has several limitations. First, the size and demographic composition of our sample may limit generalizability to broader populations and constrain our ability to fully examine the experiences of minoritized groups. In addition, some subgroup comparisons showed non‐trivial effect sizes despite non‐significant *p*‐values. These findings may reflect limited statistical power to detect between‐group differences in this sample and should therefore be interpreted cautiously. Replication in larger and more diverse samples is needed to determine whether these descriptive patterns represent stable group differences and to better capture the varied experiences and needs of different demographic groups. Second, the cross‐sectional design may not accurately capture long‐term behavioral changes needed for comprehensive analysis, and establishing causal relationships between nutritional components and clinical outcomes is difficult. For example, it remains challenging to determine whether a dietary pattern is a contributor to, or a result of, a hypertension diagnosis. Third, we used Mellen's Index to analyze the nutritional components of subgroups without identifying the specific foods consumed to meet these nutritional goals. As a result, although this approach is useful for evaluating nutrient‐based adherence, it limits our ability to determine which foods or food groups contributed to each adherence pattern and to translate findings into concrete dietary recommendations. Other indices are better able to assess dietary adherence based on consumption of food group components (Dixon et al. 2007). Future research recommended linking nutrient patterns to actual food consumption categories to provide a clearer understanding and visualization of dietary behaviors, allowing for more practical and targeted dietary recommendations. Finally, future studies should leverage longitudinal clinical trial data to evaluate whether the identified subgroups demonstrate differential responses to the intervention over time, including changes in DASH adherence and relevant clinical outcomes.

## 5 Conclusion

In this study, person‐centered approach identified distinct nutrient‐based adherence subgroups, demonstrating heterogeneity that would not be captured by a single total DASH score alone. Differences in intuitive eating subscales across subgroups suggest that DASH adherence may be shaped by distinct dietary behavior profiles, with implications for tailored intervention. These findings support the use of subgroup‐based assessment to inform more targeted and practical dietary strategies for hypertension management.

## Author Contributions

Conceptualization: Youran Lee, Qing Yang, M.C.K. Data Management: Youran Lee, Anushka Palipana, Sandy Askew, Qing Yang. Data Analysis: Youran Lee, Qing Yang. Writing – original draft preparation: Youran Lee. Writing – review and editing: Youran Lee, Qing Yang, Melissa C. Kay, Anushka Palipana, Sandy Askew, Miriam B. BergerB, H.N.M, D.M.S, G.G.B. Supervision: Qing Yang.

## Conflicts of Interest

The authors declare no conflicts of interest.

## Supporting information

Supporting File 1

## Data Availability

The data that support the findings of this study are available from the corresponding author upon reasonable request.

## References

[nur70078-bib-0001] Al‐Salmi, N. , P. Cook , and M. S. D'souza . 2022. “Diet Adherence Among Adults With Type 2 Diabetes Mellitus: A Concept Analysis.” Oman Medical Journal 37, no. 2: e361. 35441038 10.5001/omj.2021.69 PMC8994850

[nur70078-bib-0002] Batista de Lima, M. E. , and S. Eleuteri . 2021. “Increasing Patient Motivation and Adherence to Nutritional Care: The Importance to Overcome Psychological Barriers.” In Interdisciplinary Nutritional Management and Care for Older Adults: An Evidence‐Based Practical Guide for Nurses, edited by In. Ó. G. Geirsdóttir and J. J. Bell , 135–146. Springer International Publishing. 10.1007/978-3-030-63892-4_10.

[nur70078-bib-0003] Bruce, L. J. , and L. A. Ricciardelli . 2016. “A Systematic Review of the Psychosocial Correlates of Intuitive Eating Among Adult Women.” Appetite 96: 454–472. 10.1016/j.appet.2015.10.012. 26474781

[nur70078-bib-0005] Collins, L. M. , and S. T. Lanza . 2009. Latent Class and Latent Transition Analysis: With Applications in the Social, Behavioral, and Health Sciences. John Wiley & Sons.

[nur70078-bib-0006] Dixon, L. B. , A. F. Subar , U. Peters , et al. 2007. “Adherence to the USDA Food Guide, DASH Eating Plan, and Mediterranean Dietary Pattern Reduces Risk of Colorectal Adenoma3.” Journal of Nutrition 137, no. 11: 2443–2450. 17951483 10.1093/jn/137.11.2443

[nur70078-bib-0007] Van Dyke, N. , and E. J. Drinkwater . 2022. “Intuitive Eating Is Positively Associated With Indicators of Physical and Mental Health Among Rural Australian Adults.” Australian Journal of Rural Health 30, no. 4: 468–477. 10.1111/ajr.12856. 35239235 PMC9544126

[nur70078-bib-0008] Eaton, M. , Y. Probst , T. Foster , J. Messore , and L. Robinson . 2024. “A Systematic Review of Observational Studies Exploring the Relationship Between Health and Non‐Weight‐Centric Eating Behaviours.” Appetite 199: 107361. 10.1016/j.appet.2024.107361. 38643903

[nur70078-bib-0009] Epstein, D. E. , A. Sherwood , P. J. Smith , et al. 2012. “Determinants and Consequences of Adherence to the Dietary Approaches to Stop Hypertension Diet in African‐American and White Adults With High Blood Pressure: Results From the Encore Trial.” Journal of the Academy of Nutrition and Dietetics 112, no. 11: 1763–1773. 10.1016/j.jand.2012.07.007. 23000025 PMC3483427

[nur70078-bib-0010] Filippou, C. D. , C. P. Tsioufis , C. G. Thomopoulos , et al. 2020. “Dietary Approaches to Stop Hypertension (Dash) Diet and Blood Pressure Reduction in Adults With and Without Hypertension: A Systematic Review and Meta‐Analysis of Randomized Controlled Trials.” Advances in Nutrition 11, no. 5: 1150–1160. 10.1093/advances/nmaa041. 32330233 PMC7490167

[nur70078-bib-0011] Fuchs, F. D. , and P. K. Whelton . 2020. “High Blood Pressure and Cardiovascular Disease.” Hypertension 75, no. 2: 285–292. 10.1161/hypertensionaha.119.14240. 31865786 PMC10243231

[nur70078-bib-0012] Fung, T. T. 2008. “Adherence to a DASH‐Style Diet and Risk of Coronary Heart Disease and Stroke in Women.” Archives of Internal Medicine 168, no. 7: 713–720. 18413553 10.1001/archinte.168.7.713

[nur70078-bib-0013] Guo, R. , N. Li , R. Yang , et al. 2021. “Effects of the Modified DASH Diet on Adults With Elevated Blood Pressure or Hypertension: A Systematic Review and Meta‐Analysis.” Frontiers in Nutrition 8: 725020. 10.3389/fnut.2021.725020. 34557511 PMC8452928

[nur70078-bib-0014] Hensley‐Hackett, K. , J. Bosker , A. Keefe , et al. 2022. “Intuitive Eating Intervention and Diet Quality in Adults: A Systematic Literature Review.” Journal of Nutrition Education and Behavior 54, no. 12: 1099–1115. 10.1016/j.jneb.2022.08.008. 36274010

[nur70078-bib-0015] Jackson, A. , Y. Sano , L. Parker , A. E. Cox , and J. Lanigan . 2022. “Intuitive Eating and Dietary Intake.” Eating behaviors 45: 101606. 10.1016/j.eatbeh.2022.101606. 35231798

[nur70078-bib-0016] Julián‐Serrano, S. , J. Reedy , K. Robien , and R. Stolzenberg‐Solomon . 2022. “Adherence to 5 Diet Quality Indices and Pancreatic Cancer Risk in a Large US Prospective Cohort.” American Journal of Epidemiology 191, no. 9: 1584–1600. 10.1093/aje/kwac082. 35474368 PMC9989353

[nur70078-bib-0018] Kwan, M. W. M. , M. C. S. Wong , H. H. X. Wang , et al. 2013. “Compliance With the Dietary Approaches to Stop Hypertension (DASH) Diet: A Systematic Review.” PLoS One 8, no. 10: e78412. 10.1371/journal.pone.0078412. 24205227 PMC3813594

[nur70078-bib-0019] Lanza, S. T. , and B. L. Rhoades . 2013. “Latent Class Analysis: An Alternative Perspective on Subgroup Analysis in Prevention and Treatment.” Prevention Science 14, no. 2: 157–168. 10.1007/s11121-011-0201-1. 21318625 PMC3173585

[nur70078-bib-0021] Lewis, M. , L.‐M. Herron , M. D. Chatfield , et al. 2023. “Healthy Food Prices Increased More Than the Prices of Unhealthy Options During the COVID‐19 Pandemic and Concurrent Challenges to the Food System.” International Journal of Environmental Research and Public Health 20, no. 4: 3146. https://www.mdpi.com/1660-4601/20/4/3146. 36833837 10.3390/ijerph20043146 PMC9967271

[nur70078-bib-0022] Lopez, T. D. , D. Hernandez , S. Bode , and T. Ledoux . 2023. “A Complex Relationship Between Intuitive Eating and Diet Quality Among University Students.” Journal of American College Health 71, no. 9: 2751–2757. 10.1080/07448481.2021.1996368. 34788570

[nur70078-bib-0023] Lutski, M. , A. H. Stark , R. Dichtiar , S. Y. Lubel , E. M. Ornan , and T. Sinai . 2025. “Nutrition Facts Label Use and Adherence to the DASH Dietary Pattern: Results From a National Health and Nutrition Survey.” Preventing Chronic Disease 22: 240426. E31. 10.5888/pcd22.240426. PMC12249307 40576374

[nur70078-bib-0024] Marijn Stok, F. , B. Renner , J. Allan , et al. 2018. “Dietary Behavior: An Interdisciplinary Conceptual Analysis and Taxonomy.” Frontiers in Psychology 9: 1689. 10.3389/fpsyg.2018.01689. 30298030 PMC6160746

[nur70078-bib-0025] Mellen, P. B. , S. K. Gao , M. Z. Vitolins , and D. C. Goff . 2008. “Deteriorating Dietary Habits Among Adults With Hypertension: DASH Dietary Accordance, NHANES 1988‐1994 and 1999‐2004.” Archives of Internal Medicine 168, no. 3: 308–314. 18268173 10.1001/archinternmed.2007.119

[nur70078-bib-0026] Miller, H. N. , S. Askew , M. B. Berger , et al. 2025. “Effects of a Digital Intervention to Improve DASH and Blood Pressure Among US Adults.” Hypertension 82, no. 2: 370–381. 10.1161/HYPERTENSIONAHA.124.23887. 39711367 PMC11796431

[nur70078-bib-0027] Miller, H. N. , M. B. Berger , S. Askew , et al. 2021. “The Nourish Protocol: A Digital Health Randomized Controlled Trial to Promote the Dash Eating Pattern Among Adults With Hypertension.” Contemporary Clinical Trials 109: 106539. 10.1016/j.cct.2021.106539. 34400362 PMC8556291

[nur70078-bib-0029] Miller, P. E. , A. J. Cross , A. F. Subar , et al. 2013. “Comparison of 4 Established Dash Diet Indexes: Examining Associations of Index Scores and Colorectal Cancer.” American Journal of Clinical Nutrition 98, no. 3: 794–803. 10.3945/ajcn.113.063602. 23864539 PMC3743737

[nur70078-bib-0030] National Cancer Institute . (2025). Automated Self‐Administered 24‐hour Dietary Assessment Tool (ASA24). https://dceg.cancer.gov/research/how-we-study/exposure-assessment/automated-self-administered-24-hour-dietary-assessment-tool-asa24.

[nur70078-bib-0031] National Institute on Aging . (2022). Healthy Eating As You Age: Know Your Food Groups. https://www.nia.nih.gov/health/healthy-eating-nutrition-and-diet/healthy-eating-you-age-know-your-food-groups.

[nur70078-bib-0032] Ndanuko, R. N. , L. C. Tapsell , K. E. Charlton , E. P. Neale , and M. J. Batterham . 2017. “Associations Between Dietary Patterns and Blood Pressure in a Clinical Sample of Overweight Adults.” Journal of the Academy of Nutrition and Dietetics 117, no. 2: 228–239. 10.1016/j.jand.2016.07.019. 27666380

[nur70078-bib-0033] Odoms‐Young, A. , and M. A. Bruce . 2018. “Examining the Impact of Structural Racism on Food Insecurity: Implications for Addressing Racial/Ethnic Disparities.” Family & Community Health 41 Suppl 2 Suppl, Food Insecurity and Obesity, no. Suppl 2 FOOD INSECURITY AND OBESITY: S3–S6. 10.1097/fch.0000000000000183. 29461310 PMC5823283

[nur70078-bib-0034] Ostchega, Y. , C. D. Fryar , T. Nwankwo , and D. T. Nguyen . 2020. “Hypertension Prevalence Among Adults Aged 18 and Over: United States, 2017‐2018.” NCHS Data Brief 364: 1–8. 32487290

[nur70078-bib-0035] Roberts, S. B. , and I. Rosenberg . 2006. “Nutrition and Aging: Changes in the Regulation of Energy Metabolism With Aging.” Physiological Reviews 86, no. 2: 651–667. 10.1152/physrev.00019.2005. 16601270

[nur70078-bib-0037] Shang, X. , J. Liu , Z. Zhu , et al. 2023. “Healthy Dietary Patterns and the Risk of Individual Chronic Diseases in Community‐Dwelling Adults.” Nature Communications 14, no. 1: 6704. 10.1038/s41467-023-42523-9. PMC10593819 37872218

[nur70078-bib-0038] Sire, T. , N. Carbonneau , S. Lemieux , and É. Carbonneau . 2025. “Associations Between Intuitive Eating, Overall Diet Quality, and Physical Health Indicators: Results of the Predise Study.” Appetite 207: 107904. 10.1016/j.appet.2025.107904. 39929367

[nur70078-bib-0039] Soltani, S. , T. Arablou , A. Jayedi , and A. Salehi‐Abargouei . 2020. “Adherence to the Dietary Approaches to Stop Hypertension (DASH) Diet in Relation to All‐Cause and Cause‐Specific Mortality: A Systematic Review and Dose‐Response Meta‐Analysis of Prospective Cohort Studies.” Nutrition Journal 19, no. 1: 37. 10.1186/s12937-020-00554-8. 32321528 PMC7178992

[nur70078-bib-0040] Subar, A. F. , S. I. Kirkpatrick , B. Mittl , et al. 2012. “The Automated Self‐Administered 24‐hour Dietary Recall (ASA24): a Resource for Researchers, Clinicians, and Educators From the National Cancer Institute.” Journal of the Academy of Nutrition and Dietetics 112, no. 8: 1134–1137. 10.1016/j.jand.2012.04.016. 22704899 PMC3721511

[nur70078-bib-0041] Tan, J. , C. Wang , and A. J. Tomiyama . 2024. “Dietary Approaches to Stop Hypertension (Dash) Diet and Mental Well‐Being: A Systematic Review.” Nutrition Reviews 82, no. 1: 60–75. 10.1093/nutrit/nuad038. PMC10711436 37085157

[nur70078-bib-0051] Teas, E. , J. Kimiecik , R. M. Ward , and K. L. Timmerman . 2022. “Intuitive Eating and Biomarkers Related to Cardiovascular Disease in Older Adults.” Journal of Nutrition Education and Behavior 54, no. 5: 412–421. 10.1016/j.jneb.2022.01.010. 35534099 PMC9097336

[nur70078-bib-0042] Theodoridis, X. , M. Chourdakis , L. Chrysoula , et al. 2023. “Adherence to the Dash Diet and Risk of Hypertension: A Systematic Review and Meta‐Analysis.” Nutrients 15, no. 14: 3261. 10.3390/nu15143261. 37513679 PMC10383418

[nur70078-bib-0043] Theodoridis, X. , A. Triantafyllou , L. Chrysoula , et al. 2023. “Impact of the Level of Adherence to the Dash Diet on Blood Pressure: A Systematic Review and Meta‐Analysis.” Metabolites 13, no. 8: 924. 37623868 10.3390/metabo13080924 PMC10456469

[nur70078-bib-0044] Tylka, T. L. 2006. “Development and Psychometric Evaluation of a Measure of Intuitive Eating.” Journal of Counseling Psychology 53: 226–240.

[nur70078-bib-0045] Tylka, T. L. , and A. M. Kroon Van Diest . 2013. “The Intuitive Eating Scale‐2: Item Refinement and Psychometric Evaluation With College Women and Men.” Journal of Counseling Psychology 60, no. 1: 137–153. 10.1037/a0030893. 23356469

[nur70078-bib-0046] Tyson, C. C. , L. P. Svetkey , P. H. Lin , et al. 2023. “Self‐Perceived Barriers and Facilitators to Dietary Approaches to Stop Hypertension Diet Adherence Among Black Americans With Chronic Kidney Disease: A Qualitative Study.” Journal of Renal Nutrition 33, no. 1: 59–68. 10.1053/j.jrn.2022.05.002. 35597318 PMC10344422

[nur70078-bib-0047] U.S. Department of Agriculture . (2020). Dietary Guidelines for Americans, 2020–2025 https://www.dietaryguidelines.gov/sites/default/files/2020-12/Dietary_Guidelines_for_Americans_2020-2025.pdf.

[nur70078-bib-0048] Weller, B. E. , N. K. Bowen , and S. J. Faubert . 2020. “Latent Class Analysis: A Guide to Best Practice.” Journal of Black Psychology 46, no. 4: 287–311. 10.1177/0095798420930932.

[nur70078-bib-0049] Writing Committee, M. , D. W. Jones , K. C. Ferdinand , et al. 2025. “2025 AHA/ACC/AANP/AAPA/ABC/ACCP/ACPM/AGS/AMA/ASPC/NMA/PCNA/SGIM Guideline for the Prevention, Detection, Evaluation and Management of High Blood Pressure in Adults: A Report of the American College of Cardiology/American Heart Association Joint Committee on Clinical Practice Guidelines.” Hypertension (Dallas, Tex.: 1979) 82, no. 10: 212. 10.1161/HYP.0000000000000249. 40811516

