# Supplementary material for: Identifying DASH Adherence Patterns and Their Association With Dietary Behaviors Among Adults With Hypertension
Source: Res Nurs Health. 2026 May 4;49(5):553–63. doi: 10.1002/nur.70078 (PMC13539673; doi:10.1002/nur.70078)
Supplement: Supplementary file 1 [file NUR-49-553-s001.docx]

**Supplementary Table A. Mellen’s DASH target guidelines for each dietary component**

| **Nutrition components** | **Intermediate adherence**  **DASH score target** | **Fully adherent**  **DASH score target** |
| --- | --- | --- |
| **Calories** |  |  |
| **Total fat*** | 32% of calories | 27% of calories |
| **Saturated fat*** | 11% of calories | 6% of calories |
|  |  |  |
| **Sodium*** | 1286mg/1000kcal | 1143mg/1000kcal |
| **Fiber** | 9.5g/ 1000kcal | 14.8g/1000kcal |
| **Protein** | 16.5% of calories | 18% of calories |
| **Cholesterol*** | 107.1mg/1000kcal | 71.4mg/1000kcal |
| **Calcium** | 402mg/1000kcal | 590mg/1000kcal |
| **Magnesium** | 158mg/1000kcal | 238mg/1000kcal |
| **Potassium** | 1534mg/1000kcal | 2238mg/1000kcal |

*: Lower level means better adherence
